# Epstein-Barr Virus LMP1 Promotes Syntenin-1- and Hrs-Induced Extracellular Vesicle Formation for Its Own Secretion To Increase Cell Proliferation and Migration

**DOI:** 10.1128/mBio.00589-20

**Published:** 2020-06-16

**Authors:** Dingani Nkosi, Li Sun, Leanne C. Duke, Nilkumar Patel, Sunil K. Surapaneni, Mandip Singh, David G. Meckes

**Affiliations:** a Department of Biomedical Sciences, Florida State University College of Medicine, Tallahassee, Florida, USA; b College of Pharmacy and Pharmaceutical Sciences, Florida A&M University, Tallahassee, Florida, USA; UNC Chapel Hill; University of North Carolina, Chapel Hill

**Keywords:** exosomes, LMP1, Syntenin-1, Epstein-Barr virus, trafficking, extracellular vesicles, herpesvirus, ESCRT, microvesicles, oncosomes

## Abstract

Extracellular vesicles (EVs) are important mediators of cell-to-cell communication that are involved in both normal processes and pathological conditions. Latent membrane protein 1 (LMP1) is a major viral oncogene that is expressed in most Epstein-Barr virus (EBV)-associated cancers and secreted in EVs. LMP1-modified EVs have the ability to influence recipient cell growth, migration, and differentiation and regulate immune cell function. Despite the significance of LMP1-modified EVs in EBV malignancies, very little is understood about how this protein hijacks the host EV pathway for secretion. Using the biotin identification (BioID) method, we identified LMP1-proximal interacting proteins that are known to play roles in EV formation and protein trafficking. Analysis of the identified LMP1-interacting proteins revealed an enrichment in the ESCRT pathway and associated proteins, including CD63, Syntenin-1, Alix, TSG101, Hrs, and charged multivesicular body proteins (CHMPs). LMP1 transcriptionally upregulated and increased the protein expression of EV biogenesis and secretion genes. Nanoparticle tracking and immunoblot analysis revealed reduced levels of LMP1 EV packaging and of vesicle production following the knockdown of Syntenin-1, Alix, Hrs, and TSG101, with altered endolysosomal trafficking observed when Syntenin-1 and Hrs expression was reduced. Knockdown of specific ESCRT-III subunits (CHMP4B, -5, and -6) impaired LMP1 packaging and secretion into EVs. Finally, we demonstrate that the efficient secretion of LMP1-modified EVs promotes cell attachment, proliferation, and migration and tumor growth. Together, these results begin to shed light on how LMP1 exploits host ESCRT machinery to direct the incorporation of the viral oncoprotein into the EV pathway for secretion to alter the tumor microenvironment.

## Abstract (precis)

LMP1 is a notable viral protein that contributes to the modification of EV content and tumor microenvironment remodeling. LMP1-modified EVs enhance tumor proliferation, migration, and invasion potential and promote radioresistance. Currently, the mechanisms surrounding LMP1 incorporation into the host EV pathways are not well understood. This study revealed that LMP1 utilizes Hrs, Syntenin-1, and specific components of the ESCRT-III complex for release from the cell, enhancement of EV production, and metastatic properties of cancer cells. These findings begin to unravel the mechanism of LMP1 EV trafficking and may provide new targets to control EBV-associated cancers.

## INTRODUCTION

Extracellular vesicles (EVs) are virus-sized nanoparticles released from cells that are significant mediators of cell-to-cell communication in healthy and pathological environments through the delivery of biologically active molecular cargo. EVs have been shown to package and transfer specific proteins, DNA fragments, mRNAs, microRNAs (miRNAs), and lipids to neighboring or distant cells; however, there is currently only a limited understanding of the mechanisms driving cargo sorting and EV biogenesis (1, 2). Importantly, EVs have been found in almost all body fluids, which provides huge potential and opportunities for diagnostic and therapeutic applications. In malignancy, EVs play a major role in cell growth, invasion, and metastasis. In the case of virally infected cells, viruses have been shown to hijack the host endosomal machinery for viral assembly and can even modify the cargo of EVs for delivery to uninfected cells, which can enhance viral dissemination and pathogenesis (3).

The human tumor virus Epstein-Barr virus (EBV) persistently infects over 90% of the world’s population and is a major contributing factor in the development of many epithelial and lymphoid cancers (4). It is estimated that EBV accounts for roughly 200,000 new cancers each year and approximately 2% of the worldwide cancer burden (5, 6). EVs released from EBV-infected cancer cells likely contribute to tumor growth and the progression of disease. For example, latent membrane protein 1 (LMP1), an EBV oncoprotein, has been shown to enhance the secretion of EVs from infected cells (7, 8). The transfer of LMP1-containing EVs can activate mitogen-activated protein kinase (MAPK)/extracellular signal-regulated kinase (ERK) and phosphatidylinositol 3-kinase (PI3K)/Akt growth signaling functions through paracrine or autocrine mechanisms (9). LMP1-modified EVs enhance tumor cell proliferation, migration, and invasion potential and promote radioresistance of nasopharyngeal carcinoma (NPC) (10–13). Together, these data suggest that LMP1 is an important viral protein that contributes to tumor microenvironment remodeling through the transport of virally modified EVs leading to tumor growth, immune cell regulation, and cell migration and invasion. Despite the importance of LMP1-modified EVs in EBV-associated cancers, there is a limited understanding of the mechanisms responsible for orchestrating the trafficking of this viral protein into the host EV pathway.

Endosomal sorting complex required for transport (ESCRT)-dependent and ESCRT-independent cellular machineries have been found to regulate biogenesis, protein-cargo trafficking, and vesicle budding of EVs. The ESCRT complex is composed of ESCRT-0, -I, -II, and -III components, which associate with the accessory proteins Alix and VPS4. ESCRT-0, -I, and -II are involved in sequestering ubiquitinylated cargo and directing membrane budding away from the cytosol. ESCRT-III cleaves the bud necks from their cytosolic face (14–16). ESCRT machinery has also been implicated in the formation of enveloped viruses, and different viruses utilize host EV pathways to mediate intercellular communication (17). Mass spectrometry-based proteomics and lipidomics analyses of purified EVs from different cell types have been useful in identifying several ESCRT component proteins as part of the EV molecular composition (18, 19). Furthermore, a candidate (RNA interference [RNAi]) screen targeting 23 different components of the ESCRT machinery and associated proteins in HeLa CIITA cells expressing major histocompatibility complex class II (MHCII) revealed that the knockdown of Hrs, STAM1, or TSG101 reduced the secretion of EV-associated CD63 and MHCII. However, each gene knockdown differently altered the size and/or protein composition of secreted EVs (20). Alix, an ESCRT-associated protein, interacts with other ESCRT components such as TSG101 and CHMP4 for budding and abscission of the membrane (21). Furthermore, Alix is involved in EV biogenesis and cargo sorting of syndecans by binding to Syntenin-1. Syntenin-1 interacts with Alix through LYPX_n_L motifs that resemble the late assembly domains utilized by enveloped viruses to egress from cells by budding (22).

An ESCRT-independent mechanism of protein packaging and EV biogenesis has been described that requires sphingomyelinase, an enzyme that produces ceramide for the budding of intraluminal vesicles, possibly through lipid rafts or tetraspanin-enriched microdomains (23). Tetraspanin-enriched proteins such as CD9, CD63, and CD81, which are normally used as EV biomarkers, have been proposed to aid in ESCRT-dependent and -independent vesicle biogenesis depending on the cell line under investigation (24, 25). CD63 co-accumulates in vesicle populations with syndecan, Syntenin-1, and Alix, suggesting a potential role for these interactions in the formation of a unique population of EVs of a likely endosomal origin (i.e., exosomes) (22). The LMP1-CD63 association has been demonstrated to be important for LMP1 EV trafficking and LMP1-mediated upregulation of exosome biogenesis (7, 8). CRISPR/Cas9 knockout of CD63 significantly impairs the EV secretion of LMP1, which leads to downstream intracellular NF-κB overstimulation, likely due to the accumulation of LMP1 within cells (7). Together, these data suggest that the recruitment of CD63 is critical for LMP1 EV trafficking and LMP1-mediated enhancement of EV production. Since CD63 has been proposed to utilize Syntenin-1 and Alix to associate with ESCRT machinery for EV release, we sought to test the hypothesis that LMP1 similarly utilizes ESCRT-associated proteins for sorting into EVs and release from the cell.

## RESULTS

### Analysis of LMP1-interacting proteins enriched in vesicle-mediated transport.

**FIG 1:**
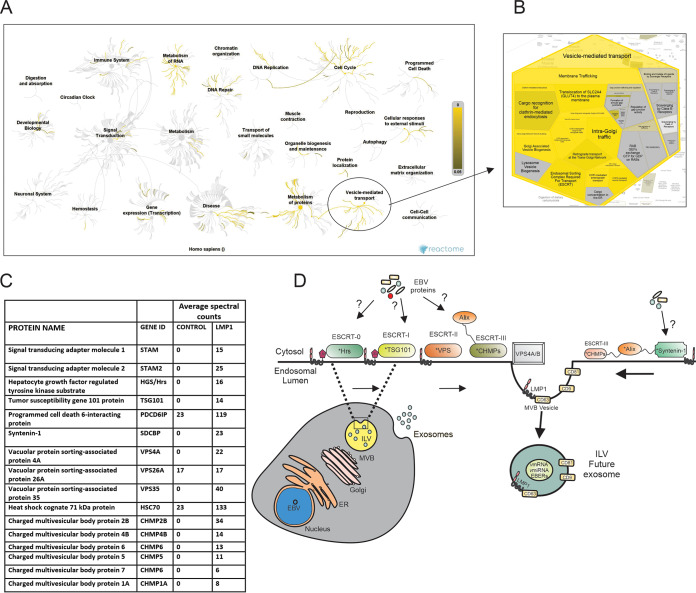
Analysis of LMP1-interacting proteins enriched in vesicle-mediated transport. All unique LMP1-interacting proteins identified were analyzed using Reactome V 69. (A) Genome-wide overview of the pathway analysis. The center of the circular “bursts” is the root of one top-level pathway, and the color-code denotes overrepresentation of that pathway in the input data set. Light gray signifies pathways that are not significantly overrepresented. (B) Representation of different downstream pathways associated with the “vesicle-mediated transport” node. (C) LMP1-interacting proteins associated with the ESCRT pathway. (D) Model of different ESCRT components involved in extracellular vesicle formation and secretion. ESCRT components with * in the model were identified as LMP1-interacting proteins. ER, endoplasmic reticulum; vmiRNA, viral microRNA; ILV, intraluminal vesicles; GEFs, guanine nucleotide exchange factors; COPI, coat protein I.

Using the proximity-dependent biotin identification (BioID) method, we discovered LMP1-proximal and direct interacting proteins from cells expressing BirA-LMP1 or LMP1-BirA (fusion proteins). The captured biotinylated proteins were separated using SDS-PAGE, and the fractionated proteins were subjected to trypsin digestion and mass spectrometry analyses (26). We identified over 1,200 proteins with direct, transient, or proximal associations with LMP1 (26). These data represent the larger LMP1 interaction network that is not restricted to direct interactions like the TRAF proteins (26). To begin to better understand the pathways that could be affected by LMP1, all unique LMP1-interacting proteins identified and not present in control samples were analyzed using Reactome (27). Reactome uses an overrepresentation analysis statistical test that determines if certain pathways are enriched in the submitted data, and a probability score corrected for the false discovery rate using the Benjamini-Hochberg method is provided (27). The color-code denotes an overrepresentation of that pathway in the input data set, and light gray signifies pathways that are not significantly overrepresented. Genome-wide overview analysis (Fig. 1A) revealed all the pathways associated with the LMP1-interacting proteins. The most significantly identified pathways include metabolism of RNA, metabolism of proteins, DNA repair, DNA replication, the cell cycle, and vesicle-mediated transport (Fig. 1A). The representation of different downstream pathways associated with the “vesicle-mediated transport” node includes membrane trafficking, ESCRT, intra-Golgi traffic, Golgi-associated vesicle biogenesis, and others (Fig. 1B). Some of the identified proteins are known to play key roles in exosome formation and protein trafficking, including CD63, Syntenin-1, Alix, TSG101, Hrs, charged multivesicular body proteins (CHMPs), and sorting nexins (Fig. 1C). Most of the LMP1-interacting proteins identified in the vesicle-mediated transport node are enriched in ESCRT pathway components. The ESCRT pathway and associated proteins have been shown to regulate biogenesis, protein-cargo trafficking, and vesicle budding of EVs. Therefore, we hypothesized that components of the ESCRT pathway are important for LMP1 EV incorporation and LMP1-mediated enhancement of vesicle production (Fig. 1D).

### LMP1 expression increases mRNA and protein levels of EV biogenesis components.

**FIG 2:**
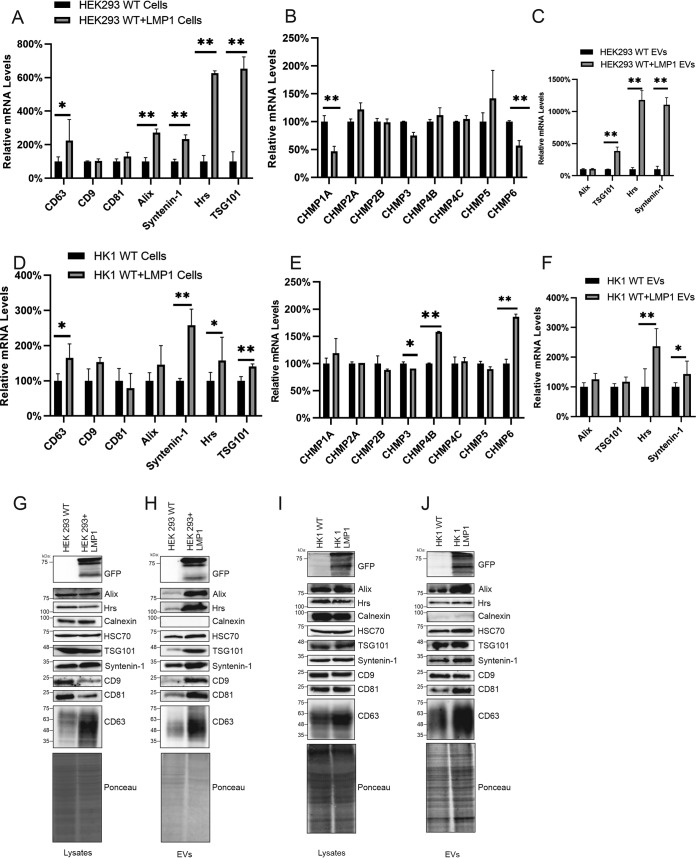
LMP1 expression increases mRNA and protein levels of EV biogenesis components. mRNA was collected from normal HEK293 cells or HEK293 cells stably expressing an inducible LMP1 construct and subjected to RT-qPCR. (A and B) Relative mRNA levels of the different EV biogenesis components (CD9, CD63, CD81, Hrs, Alix, Syntenin-1, TSG101, CHMP1A, CHMP2A, CHMP2B, CHMP3, CHMP4B, CHMP4C, CHMP5, and CHMP6) were determined by normalization to GAPDH levels (for HEK293 cells) and normal HEK293 cells (for HEK293 cells stably expressing LMP1). (C) Relative mRNA levels of different EV biogenesis genes (Hrs, Alix, Syntenin-1, and TSG101) collected from EVs of HEK293 cells or HEK293 cells expressing LMP1. (D and E) Relative mRNA levels of EV biogenesis components collected from the nasopharyngeal carcinoma cell line HK1 or HK1 expressing inducible LMP1. (F) Relative mRNA levels of different EV biogenesis genes collected from EVs of HK1 cells or HK1 cells expressing LMP1. (G to J) Immunoblot analysis of cell and vesicle lysates from wild-type HEK293 or HK1 cells and HEK293 or HK1 cells expressing inducible LMP1 showing expression levels of the different EV biogenesis components from 3 independent experiments (*, *P* < 0.05; **, *P* < 0.001).

LMP1 activates several signaling pathways, including MAPK/ERK, PI3K/Akt, NF-κB, mTOR, and c-Jun N-terminal kinase (cJNK) (29–33). These signal transductions result in the transcriptional upregulation of different genes that are involved with the regulation of apoptosis, cell cycle progression, and cell proliferation, migration, and invasion. From these data, we postulated that LMP1 transcriptionally upregulates ESCRT pathway components and its associated proteins to enhance EV production and protein trafficking. To test this, mRNA was collected from normal HEK293 cells or HEK293 cells stably expressing inducible LMP1 and subjected to real-time quantitative PCR (RT-qPCR). LMP1 increased the mRNA expression of CD63, Alix, Syntenin-1, Hrs, TSG101, and CHMP5 compared to control cells (Fig. 2A and B). Interestingly, LMP1 decreased the mRNA expression of CHMP1A and CHMP6. Furthermore, LMP1 increased mRNA sorting of Syntenin-1, Hrs, and TSG101 into EVs (Fig. 2C), suggesting that LMP1-modified EVs may enhance vesicle production in cells through paracrine or autocrine mechanisms. To increase the relevance of these results to EBV-associated cancers, Hong Kong 1 (HK1), a nasopharyngeal carcinoma cell line stably expressing inducible LMP1, was compared to HK1 wild-type (WT) cells. The results showed that LMP1 increases the mRNA expression of CD63, Syntenin-1, Hrs, TSG101, CHMP6, and CHMP4B in cells and Hrs and Syntenin-1 in EVs (Fig. 2D to F). Additionally, we assessed whether the expression of LMP1 affects the protein expression of the different EV biogenesis genes. Inducing the expression of LMP1 in HEK293 cells increased the protein expression of Syntenin-1 and CD63. Higher levels of Alix, Hrs, TSG101, Syntenin-1, HSC70, CD9, CD81, and CD63 were also found outside the cell when EVs from an equal volume of medium were analyzed (Fig. 2G and H). This is likely due to the fact that LMP1 enhances EV production and further demonstrates the elevated expression of these proteins due to LMP1. LMP1 induction in HK1 cells also enhanced the protein expression of CD63 and Syntenin-1 in cells and Alix, TSG101, Syntenin-1, HSC70, CD81, and CD63 in the EV fraction, corroborating the findings in HEK293 cells (Fig. 2I and J). The differences in LMP1-enhanced EV secretion of CD9, Hrs, and TSG101 between the two cell lines are likely due to cell type-specific differences in EV cargo sorting or production (20, 34). Altogether, our results suggest that LMP1 transcriptionally upregulates and increases the protein expression of different ESCRT components involved in the trafficking of proteins and the biogenesis of EVs. This is likely one of the ways that the viral oncoprotein utilizes to increase EV biogenesis and its own sorting and packaging into EVs.

### Knockdown of upstream ESCRT-dependent components reduces LMP1 packaging and vesicle secretion.

**FIG 3:**
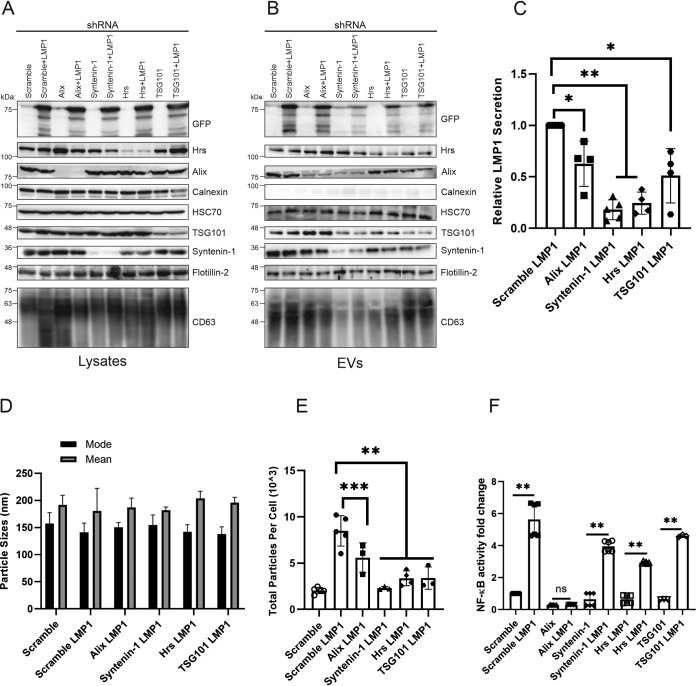
Knockdown of upstream ESCRT-dependent proteins reduces LMP1 vesicle secretion and packaging. HEK293 cells expressing inducible scramble, Alix, Hrs, Syntenin-1, or TSG101 shRNA constructs were transfected with GFP-LMP1. (A and B) Whole-cell (equal mass loaded) (A) and EV (equal volume loaded) (B) lysates were separated by SDS-PAGE and analyzed by immunoblot analysis for LMP1 and common EV markers (Alix, Hrs, calnexin, HSC70, TSG101, Syntenin-1, Flotillin-2, and CD63). (C) Semiquantitative Western blot analysis of results from more than 3 independent experiments. The data are presented relative to the values for wild-type LMP1 packaged into EVs. (D and E) EVs harvested from HEK293 cells expressing shRNAs transfected with GFP-LMP1 were analyzed by nanoparticle tracking analysis for size (D) and quantity (E). (F) HEK293 cells expressing the shRNAs and the NF-κB luciferase reporter were transfected with LMP1 and evaluated for activation of NF-κB signaling (*, adjusted *P* < 0.005; **, adjusted *P* < 0.0001; ***, adjusted *P* < 0.05; ns, not significant).

The upstream ESCRT components contain ubiquitin binding domains; the ESCRT-0 complex is responsible for sequestering ubiquitylated cargo in the endosomal membrane, while the ESCRT-I and -II complexes control membrane deformation into vesicles with sequestered cargo, leading to its release as EVs (14, 16, 35). Syntenin-1 has been found to bind Alix through the LYPX_n_L motifs or L domains, which are similar to retroviral Gag proteins used for viral budding (22, 36, 37). The Alix–Syntenin-1 interaction in conjunction with certain ESCRT components also plays a role in membrane budding and scission leading to the secretion of EVs. To determine the role of the different upstream ESCRT proteins identified in the BioID experiments in LMP1 EV trafficking, we generated HEK293 cells expressing inducible scramble, Alix, Hrs, Syntenin-1, and TSG101 short hairpin RNA (shRNA) constructs (Fig. 3A). Different cell lines harboring the shRNAs were transfected with green fluorescent protein (GFP)-tagged LMP1 to assess LMP1 EV secretion and packaging. Immunoblot analysis showed that the knockdown of Alix, Hrs, Syntenin-1, and TSG101 decreased LMP1 packaging in the EVs compared to the scrambled control (Fig. 3B). Semiquantitative analysis of Western blots revealed that the knockdown of Syntenin-1 and Hrs produced the greatest inhibition of LMP1 packaging into EVs compared to Alix and TSG101 (Fig. 3C). Interestingly, CD63 accumulated to high levels in Alix knockdown cells but not when LMP1 was expressed. These data suggest that LMP1 may be altering CD63 trafficking or protein-protein interactions, diverting it to lysosomes or autophagic vacuoles, as suggested by others (8, 38, 39). Nanoparticle tracking of the cell lines expressing the different shRNAs complemented the Western blot results in that it revealed reduced EV secretion following LMP1 transfection (Fig. 3E). The knockdown of Alix, Hrs, Syntenin-1, and TSG101 also reduced total particles secreted from the cells compared to the scramble LMP1 control, but no significant changes were noted in terms of the mode and size of the EVs (Fig. 3D and E). Further nanoparticle tracking analysis showed a mode vesicle size of around 150 nm across all groups, which is the smaller EV subpopulation where LMP1 is enriched. Although in these experiments, we did not examine distinct EV subpopulations, our results suggest that Alix, TSG101, Hrs, and Syntenin-1 are associated with sorting LMP1 into smaller EVs and may be present in the same EVs following budding and release from the cell. It is likely that these proteins are also present in other EV subpopulations with distinct cargo (40). Additionally, we evaluated the effect of knocking down different upstream ESCRT components on the activation of NF-κB signaling. To test this, the different HEK293 cells expressing inducible shRNAs were transduced with viral particles expressing an NF-κB luciferase reporter to make stable cell lines. This was followed by transfecting LMP1 in the cells. These experiments revealed that LMP1 enhanced NF-κB signaling in all groups transfected compared to the control (scramble), except in cells expressing the Alix shRNA (Fig. 3F). Taken together, our data point toward an important role for upstream ESCRT components and associated proteins in directing LMP1 into EVs and secretion from the cell and LMP1-mediated enhancement of vesicle production.

### ESCRT-III subunits decrease LMP1 packaging into extracellular vesicles.

**FIG 4:**
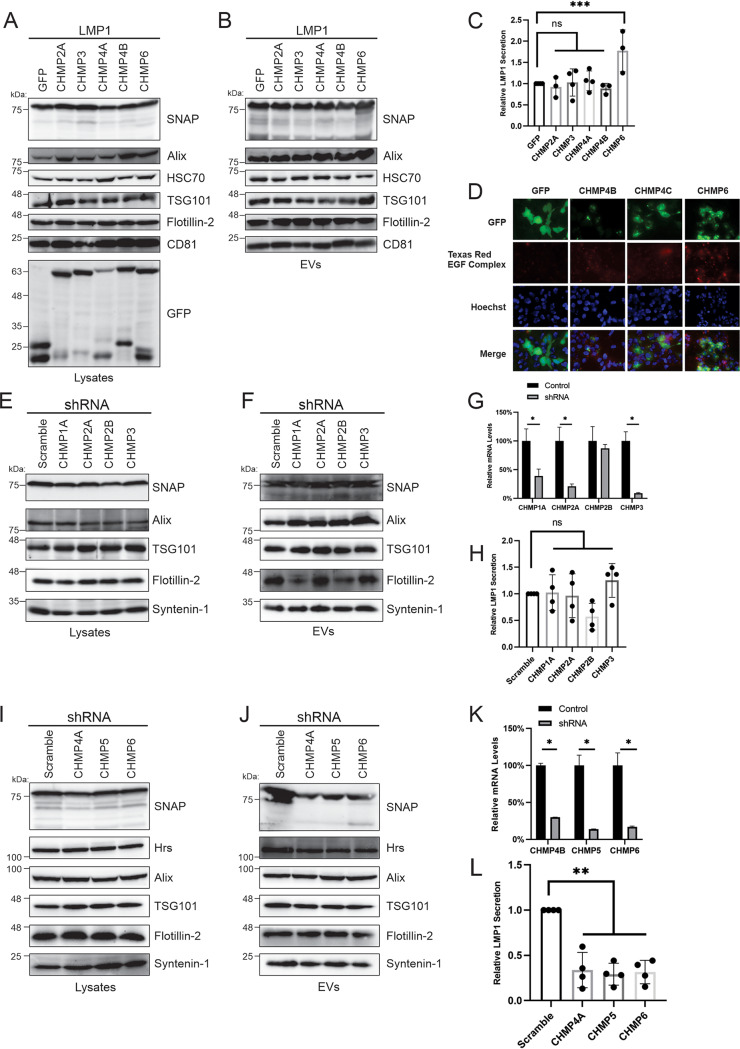
ESCRT-III subunits decrease LMP1 packaging into extracellular vesicles. HEK293 cells expressing inducible GFP as a control and GFP-tagged dominant negative constructs against the ESCRT-III subunits (CHMP2A, CHMP3, CHMP4A, CHMP4B, and CHMP6) were transfected with SNAP-LMP1. (A and B) Whole-cell (A) and EV (B) lysates were separated by SDS-PAGE and analyzed by immunoblot analysis for LMP1 and common EV markers (Alix, HSC70, TSG101, Flotillin-2, and CD81). (C) Semiquantitative Western blot analysis of results from more than three independent experiments showing LMP1 relative EV secretion for HEK293 cells expressing ESCRT-III dominant negative constructs. (D) Internalization and degradation of fluorescently labeled EGF in cells expressing dominant negative constructs against ESCRT-III dominant negative constructs. (E to L) HEK293 cells stably expressing shRNAs against ESCRT-III subunits (CHMP1A, CHMP2A, CHMP2B, CHMP3, CHMP4A, CHMP5, and CHMP6) were generated and transfected with SNAP-LMP1. (E, F, I, and J) Immunoblot analysis of cell (E and I) and vesicle (F and J) lysates from cells expressing shRNA. (G and K) Relative mRNA levels of CHMPs (CHMP1A, -2A, -2B, -3, -4A, -5, and -6) in cells stably expressing shRNAs. (H and L) Semiquantitative Western blot analysis of results from more than 3 independent experiments. Data are presented relative to the values for wild-type LMP1 packaged into EVs (*, adjusted *P* < 0.005; **, adjusted *P* < 0.0001; ***, adjusted *P* < 0.05).

The ESCRT-III complex is composed of about 12 subunits that are required for the scission of intraluminal vesicles on endosomal membranes, cytokinesis, and the budding of some viruses (16, 41, 42). The four core subunits thought to be required for forming the scission complex include CHMP6, CHMP4, CHMP3, and CHMP2 (43). To assess if the different core subunits affect LMP1 sorting into EVs, HEK293 cell lines stably expressing inducible ESCRT-III dominant negative subunits were made. GFP-tagged CHMP2A, CHMP3, CHMP4A, CHMP4B, and CHMP6 dominant negative cell lines were transfected with SNAP-tagged LMP1 (Fig. 4A). Western blot analysis revealed that the expression of the dominant negative constructs against the ESCRT-III subunits did not block LMP1 packaging into EVs (Fig. 4B). In fact, quantification of data from three independent experiments revealed a slight increase in LMP1 secretion with the CHMP6 dominant negative construct compared to the wild-type protein (Fig. 4C). CHMP2A, CHMP3, and CHMP4A dominant negative constructs displayed levels of packaging of LMP1 similar to those of the wild-type counterparts, with CHMP4B slightly reducing extracellular LMP1 levels (Fig. 4C). To validate that the cell lines expressing dominant negative constructs against ESCRT-III subunits were functional and sufficient to block endolysosomal pathways, we tested the ability of the cells to internalize and degrade fluorescently tagged epidermal growth factor (EGF). EGF is endocytosed from the plasma membrane and traffics to lysosomes for degradation through the ESCRT pathway following engagement with its receptor on the cell surface (43). Induction of the ESCRT-III dominant negative cell lines with doxycycline showed clear EGF punctae in the cells compared to the GFP sample, indicating an ability to block EGF degradation (Fig. 4D).

Due to the inability of the ESCRT-III dominant negative subunits to block LMP1 packaging into EVs, we hypothesized that the dominant negative proteins may not be effective at blocking the WT protein function once the scission complex is formed. Therefore, we generated stable HEK293 cell lines expressing shRNAs against different ESCRT-III components to inhibit complex formation or scission function. The stable cell lines exhibited effective knockdown of the ESCRT-III subunits (CHMP1A, -2A, -2B, -3, -4B, -5, and -6) targeted (Fig. 4G and K). Knockdown of the ESCRT-III subunits CHMP1A, -2A, -2B, and -3 did not inhibit the packaging of LMP1 into EVs (Fig. 4E and F). The relative level of EV secretion of LMP1 from the cells expressing these shRNAs was almost similar to the scramble levels (Fig. 4H). However, when CHMP4A, -5, and -6 were knocked down, reduced packaging of LMP1 into EVs was observed (Fig. 4I and J). Quantitative analysis of Western blots from more than three independent experiments showed that knocked-down CHMP4A, -5, and -6 had lower levels of LMP1 relative secretion than the wild type (Fig. 4L). These data suggest that distinct ESCRT-III subunits may play unique roles in aiding LMP1 packaging and sorting of LMP1 into EVs, with others being dispensable.

### Dominant negative VPS4A does not impair LMP1 packaging into EVs.

**FIG 5:**
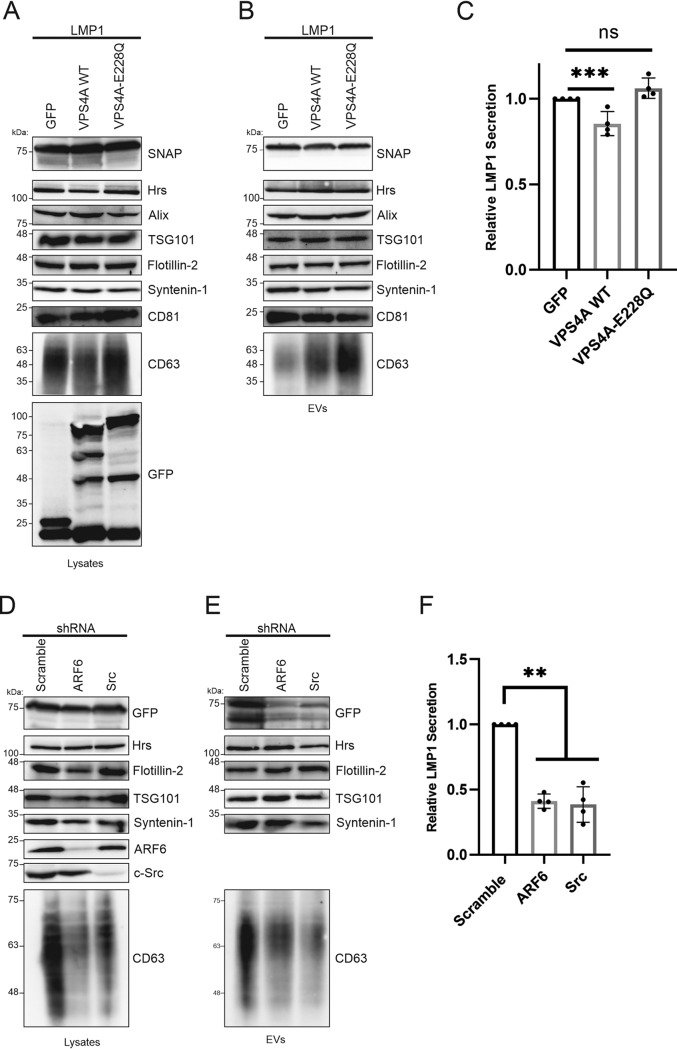
A VPS4A dominant negative construct does not impair LMP1 vesicle packaging. HEK293 cells expressing inducible GFP and GFP-tagged wild-type VPS4A (VPS4A WT) or VPS4A-E228Q, which blocks ATP hydrolysis and acts as a dominant negative construct, were transfected with SNAP-LMP1. (A and B) Immunoblot analysis of cell and vesicle lysates for cells expressing GFP, VPS4A, and the mutant VPS4A-E228Q. (C) Semiquantitative Western blot analysis of results from more than 3 independent experiments. Data are presented relative to the value for wild-type LMP1 packaged into EVs. (D to F) HEK293 cells stably expressing shRNAs against ARF6 and c-SRC were constructed and transfected with SNAP-LMP1. (D and E) Whole-cell (D) or EV (E) lysates were separated by SDS-PAGE and analyzed by immunoblot analysis for LMP1 and common EV markers. (F) Semiquantitative Western blot analysis of results from three independent experiments showing relative LMP1 EV secretion (**, adjusted *P* < 0.0001; ***, adjusted *P* < 0.05).

VPS4A is recruited by the ESCRT-III complex, catalyzes the final membrane scission step, and is important for the recycling of ESCRT-III components (43). We therefore hypothesized that mutation of the VPS4A complex might alter LMP1 sorting into EVs. To test this, we generated HEK293 cells expressing inducible GFP and GFP-tagged wild-type VPS4A (VPS4A WT) or the dominant negative mutant VPS4A-E228Q, which blocks ATP hydrolysis, and transfected them with SNAP-LMP1. Immunoblot analysis of cell and vesicle lysates demonstrated similar levels of LMP1 packaging into EVs for VPS4A and the mutant VPS4A-E228Q compared to GFP (Fig. 5A and B). Quantitation of the results showed comparable levels of LMP1 packaging between VPS4A-E228Q and the GFP-only control (Fig. 5C).

Syntenin-1-mediated small EV formation has been shown to be controlled by ARF6 and its effector PLD2 upstream of the ESCRTs (36, 37). The SRC kinase has also been identified as a key regulator of EV biogenesis through the Syntenin-1–syndecan pathway upstream of ARF6 (44). Since our data showed that knocking down Syntenin-1 could impair LMP1 sorting into EVs (Fig. 3B, C, and E), we wondered whether ARF6 or SRC could also regulate LMP1 incorporation into EVs. To test this, HEK293 cells stably expressing shRNAs against ARF6 and SRC kinase were generated and transfected with LMP1. Western blot analysis of the cell and EV lysates revealed that knocking down ARF6 or SRC reduces the packaging of LMP1 into EVs but not all EV markers (Fig. 5D to F). Interestingly, the knockdown of ARF6 and SRC dramatically affected CD63 cellular levels and EV incorporation. CD63 is packed into EVs through a Syntenin-1–syndecan–Alix pathway and was previously shown to be important for LMP1 EV packaging. Therefore, the pronounced defect in LMP1 EV incorporation with ARF6 and SRC knockdown may be due mostly to the decreased CD63 levels. It could be that CD63 acts as the bridge between LMP1 and the syntenin–syndecan–Alix pathway. Taken together, these findings suggest that LMP1 uses Syntenin-1 and the upstream SRC/ARF6 pathway to enter the ESCRT pathway for EV release.

### Syntenin-1 and Hrs knockdowns exhibit altered LMP1 endolysosomal trafficking.

**FIG 6:**
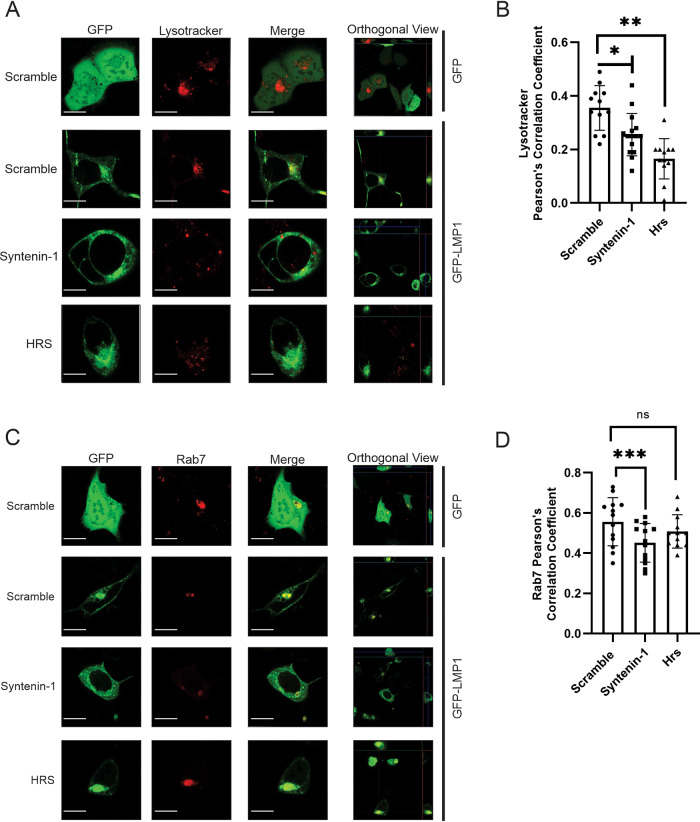
Syntenin-1 and Hrs knockdowns exhibit altered LMP1 endolysosomal trafficking. (A and C) Cells expressing shRNAs were either transfected with GFP-LMP1 and then stained with Lysotracker at 24 h posttransfection or cotransfected with GFP-LMP1 and Rab7. Live-cell confocal images were acquired at 24 h posttransfection on a Zeiss microscope. (B and D) Colocalization was quantified using Pearson’s correlation coefficient (*n* = 8 cells). Representative maximum-projection images are shown (*, adjusted *P* < 0.005; **, adjusted *P* < 0.0001; ***, adjusted *P* < 0.05). Bars, 10 μm.

LMP1 has been shown to traffic through the endocytic routes and accumulate in late endosomes, lysosomes, and multivesicular bodies (MVBs). LMP1 secretion through EVs is believed to facilitate the evasion of proteasomal or lysosomal degradation. Since Hrs or Syntenin-1 knockdown revealed a significant reduction in LMP1 sorting into EVs, we hypothesized that the LMP1 subcellular localization would be altered in the endolysosomal components of the knockdown cells compared to the cells with a scramble shRNA. To test this, the colocalization of LMP1 with the subcellular compartment markers Rab7 (late endosomes) and Lysotracker (lysosomes) was examined in HEK293 cells expressing scramble, Hrs, or Syntenin-1 shRNAs (Fig. 6A and B). The results demonstrate that LMP1 displays more colocalization with Lysotracker in control cells (Pearson correlation coefficient [PCC] = 0.38) (Fig. 6B) than Hrs (PCC = 0.16; adjusted *P* < 0.0001) or Syntenin-1 (PCC = 0.26; adjusted *P* = 0.0049) knockdown cells. Surprisingly, LMP1 colocalization with Rab7 was similar in control cells (PCC = 0.55) and Hrs (PCC = 0.51; adjusted *P* value of 0.4024)-knocked-down cells compared to the Syntenin-1 (PCC = 0.45; adjusted *P* value of 0.0237) shRNA-expressing cells, which exhibited less colocalization (Fig. 6D). Taken together, our data suggest that Hrs and Syntenin-1 regulate LMP1 endolysosomal trafficking.

### Knockdown of Syntenin-1 and Hrs in the nasopharyngeal carcinoma cell line HK1 impairs LMP1 vesicle packaging and secretion.

**FIG 7:**
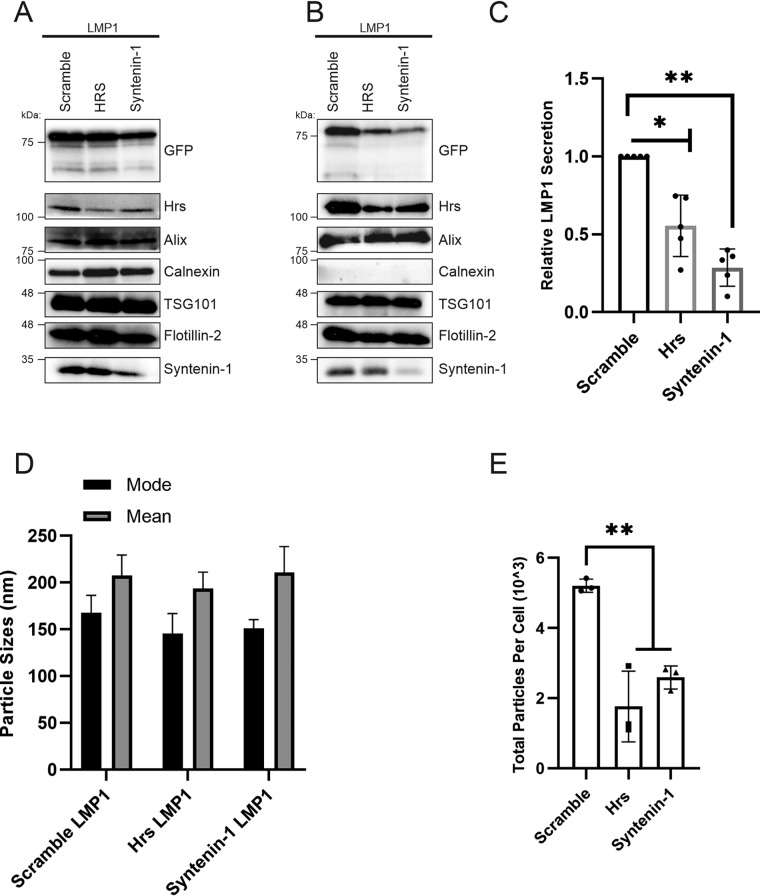
Knockdown of Syntenin-1 and Hrs in the nasopharyngeal carcinoma cell line HK1 impairs LMP1 vesicle secretion and packaging. HK1 cells expressing inducible shRNAs (scramble, Hrs, and Syntenin-1) and inducible GFP-LMP1 were generated. (A and B) Whole-cell (A) and EV (B) lysates were separated by SDS-PAGE and analyzed by immunoblot analysis for LMP1 and common EV markers (Alix, Hrs, calnexin, HSC70, TSG101, Syntenin-1, Flotillin-2, and CD63). (C) Semiquantitative Western blot analysis of results from more than 3 independent experiments. The data are presented relative to the value for wild-type LMP1 packaged into EVs. (D and E) Nanoparticle tracking analysis for size (D) and quantity (E). (*, adjusted *P* < 0.005; **, adjusted *P* < 0.0001).

To further understand the role of the ESCRT pathway and associated proteins in LMP1 packaging into EVs in the context of an EBV-associated cancer cell line, inducible HK1 cells expressing LMP1 were transduced with lentiviral particles containing Hrs, Syntenin-1, or scramble shRNAs. Following transduction, the cells underwent selection to generate a population of cells stably expressing both inducible LMP1 and the shRNAs. Immunoblot analysis demonstrated that knocking down Hrs or Syntenin-1 in HK1 cells also reduced LMP1 packaging into EVs (Fig. 7A and B). Quantitative analysis of the Western blots showed reduced LMP1 secretion when Hrs or Syntenin-1 was knocked down compared to the scramble (Fig. 7C). Again, nanoparticle tracking analysis revealed that Hrs and Syntenin-1 contribute to the LMP1-mediated enhancement of vesicle production (Fig. 7D and E). Taken together, these results show that LMP1 is likely utilizing Hrs and Syntenin-1 for incorporation into EVs for secretion and promoting EV biogenesis in NPC.

### LMP1 secretion into EVs enhances cell proliferation and migration and tumor growth.

**FIG 8:**
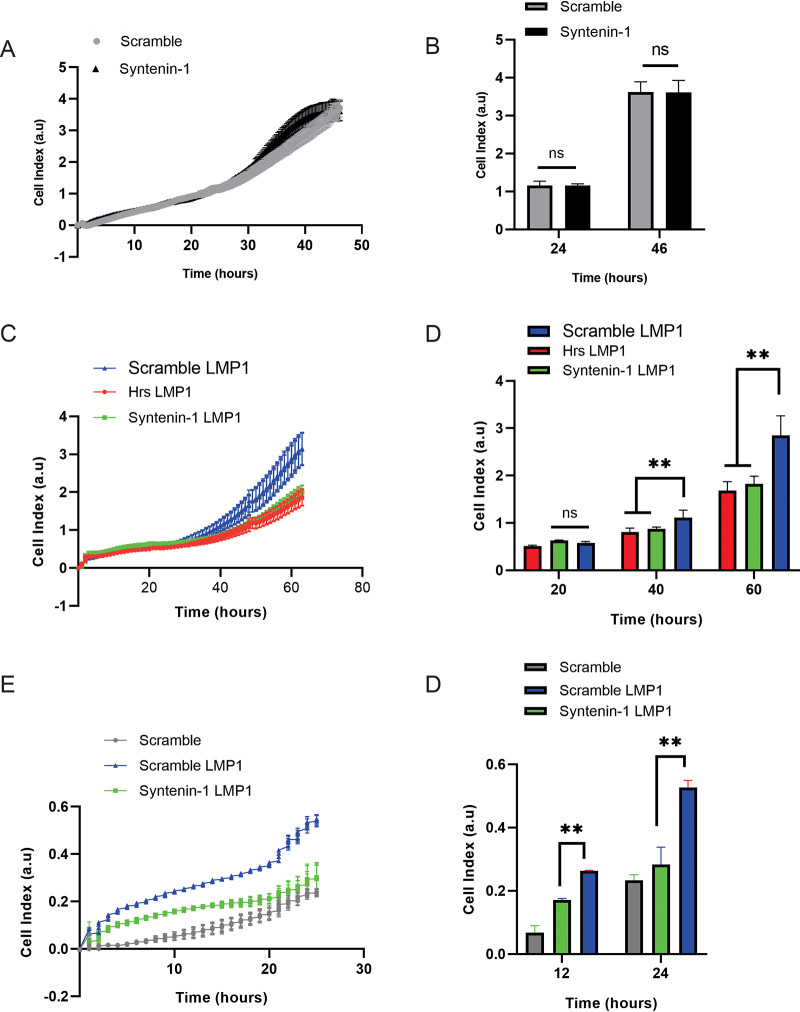
Efficient LMP1 EV secretion enhances cell proliferation and migration and tumor growth. (A) HK1 cells expressing scramble shRNA had the same cell growth and proliferation as HK1 cells expressing Syntenin-1 shRNA. HK1 cells expressing the inducible shRNAs (scramble and Syntenin-1) were plated in an xCelligence E-16 plate, and growth was monitored for about 60 h. (B and C) HK1 cells expressing inducible LMP1 accelerate cell proliferation and growth. (D and E) LMP1 EV secretion promotes cell migration. HK1 cells expressing inducible LMP1 and shRNAs (scramble and Syntenin-1) were plated in an xCelligence CIM-16 plate, and cell migration was monitored for 24 h (**, adjusted *P* < 0.0001). a.u, arbitrary units.

To investigate the role of LMP1-modified EVs in cell proliferation and migration, we utilized the xCelligence system, which can monitor such properties. The xCelligence system uses noninvasive electrical impedance to monitor different cell phenotypes in real time. The cell growth and proliferation of control cells expressing scramble and Syntenin-1 shRNAs only were initially compared and found to be similar (Fig. 8A and B). Cell growth and proliferation were also assessed in HK1 cells expressing either scramble, Hrs, or Syntenin-1 shRNA plus LMP1. LMP1 expression in HK1 control cells increased cell proliferation and growth compared to those when Hrs and Syntenin-1 were knocked down in cells (Fig. 8C and D). We furthermore evaluated whether the efficient secretion of LMP1-modified EVs would act as a chemoattractant and facilitate cell migration. The efficient secretion of LMP1 into EVs increased cell migration compared to EVs produced in Syntenin-1 knockdown cells (Fig. 8E and F). Overall, these experiments begin to demonstrate the important role of LMP1-modified EVs in enhancing tumorigenesis and remodeling the tumor microenvironment.

### Efficient secretion of EV-associated LMP1 promotes tumor growth.

**FIG 9:**
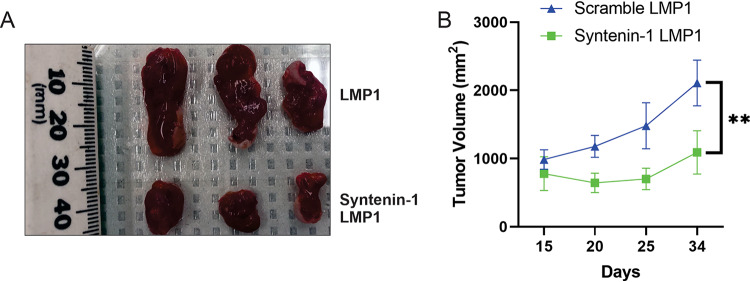
Efficient secretion of LMP1-modified EVs promotes tumor formation and growth. HK1 cells expressing inducible shRNAs (scramble and Syntenin-1) and LMP1 were injected into mice, and tumor growth was monitored for about 30 days. More than 3 mice were used for each group (**, adjusted *P* < 0.0001).

To verify the role of efficient secretion of LMP1-containing EVs in tumor formation and growth, animal xenograft studies were conducted. HK1 cells expressing LMP1 only or LMP1 and Syntenin-1 shRNA were injected into athymic nude mice, and tumor growth was monitored for about 30 days. Our results indicated that tumor growth was inhibited when efficient sorting of LMP1 was blocked through Syntenin-1 knockdown (Fig. 9A and B). These results support a major role for LMP1-modified EVs in promoting tumor growth and also highlight the pathways that the viral oncoprotein might be using for secretion into the extracellular space. However, we cannot rule out the possibility that Syntenin-1 may also affect tumor growth in the absence of LMP1 or through other mechanisms not due to LMP1 EV secretion.

## DISCUSSION

Mechanisms surrounding LMP1 sorting into EVs and its secretion are still not well understood. In this study, we investigated the role of the ESCRT pathway and associated proteins in LMP1-enhanced EV packaging, secretion, and release from cells. Multiple studies have shown how different viruses hijack the ESCRT pathway for virus budding. Late domains (PPXY and PTAP) interact with ESCRT components to initiate the budding of nascent virions (16, 42, 45). Here, we demonstrate that the LMP1-interacting proteins Hrs and Syntenin-1 play major roles in directing LMP1 into EVs for packaging and secretion. Syntenin-1 and Hrs knockdown revealed a significant decrease in LMP1 packaging into EVs and exhibited altered endolysosomal trafficking. Likewise, the knockdown of the ESCRT-III components CHMP4A, -5, and -6 also decreased LMP1 EV packaging and secretion. Furthermore, the knockdown of Syntenin-1 in a nasopharyngeal carcinoma cell line expressing LMP1 reduced EV secretion, EV packaging, cell proliferation and migration, and tumor growth. Taken together, these findings reveal for the first time the importance of Syntenin-1 and components of the ESCRT pathway for LMP1 EV trafficking.

The ESCRT pathway and its associated proteins have been shown to be one of the major machineries responsible for EV biogenesis and cargo sorting leading to EV release from the cell (20, 46, 47). Bioinformatic, mRNA, and protein expression analyses revealed an enrichment in ESCRT pathway proteins, including CD63, Syntenin-1, Alix, TSG101, Hrs, charged multivesicular body proteins (CHMPs), and sorting nexins (Fig. 1C). Some of the identified proteins were previously verified by immunoprecipitation to interact with LMP1 (26). The transcriptional upregulation of these EV biogenesis genes may be a result of the activation of several signaling pathways by LMP1 (31, 48–50). The increased expression of proteins involved in EV biogenesis and secretion following LMP1 induction might also be acting as a cellular response to the intracellular accumulation of LMP1 triggering different signaling cascades. We previously demonstrated that blocking LMP1 secretion into EVs results in the overstimulation of intracellular NF-κB within the cell. The appropriate termination of these signaling transductions through the efficient secretion of LMP1 into EVs is critical for cell health and may be important for viral persistence. Overall, these data begin to enlighten how this viral oncoprotein might be utilizing host cellular trafficking pathways to the benefit of EBV.

Hrs is part of the ESCRT-0 complex, which is on the endosomal membranes and responsible for binding ubiquitinated cargo within early endosomes (35, 42, 51). Hrs acts as endocytic sorting machinery for the transport of proteins for either lysosomal degradation or EV secretion (14, 52). LMP1 is ubiquitinated and targeted for degradation by the proteasome and lysosome, or alternatively, it is packed into intraluminal vesicles and secreted as EVs (8, 9, 39, 53, 54). Our results suggest that Hrs might be recruiting ubiquitinated LMP1 into the endosomal membranes for EV secretion or lysosomal degradation. A previous study showed that tagging the N terminus of LMP1 with a noncleavable ubiquitylated fusion protein resulted in increased sorting and secretion of nontagged LMP1 rather than ubiquitin-tagged LMP1 (8). From that study, the authors concluded that ubiquitylation does not affect LMP1 incorporation and secretion into EVs, but those studies were limited in that they did not exclude the possibility that ubiquitin links on other lysines within LMP1 could still be removed before LMP1 is sorted into EVs. Additionally, ubiquitin may be required during the initial stages of the endocytic process to interact with proteins like Hrs or STAM1. Further studies looking at the ubiquitination of LMP1 and its role in EV sorting and secretion are required. The depletion of Hrs has also been shown to decrease the number of ESCRT-I components and the number of multivesicular bodies (51, 55, 56). Our observed results might be explained by a failure to recruit the ESCRT-I components due to Hrs depletion, and hence, LMP1 is not clustered efficiently to the endosomal membranes. Furthermore, the reduced colocalization between LMP1 and Lysotracker in the Hrs knockdown compared to the scramble control may be because of the reduced number of multivesicular bodies being generated in these cells, and therefore, not much LMP1 is being targeted for degradation. Taken together, these results indicate that Hrs plays a critical role in recruiting LMP1 to endosomal membranes and then targeting it to lysosomes for degradation or to EVs for secretion. While accumulating evidence suggests that the majority of LMP1 is released from cells in small EVs derived from internal endosomal membranes of multivesicular bodies (7–9, 39, 57), it is still possible that a portion of LMP1 may also bud off the plasma membrane in the form of microvesicles (8, 57, 58). Current limitations in EV purification protocols make it difficult to obtain truly pure exosome or microvesicle preparations for biochemical analyses. It is also possible topologically that exosomes could bud from the plasma membrane following MVB fusion (59, 60). Therefore, we have chosen to refer to the EVs analyzed in this study as small EVs, as described by others (40, 61).

Syntenin-1 has been implicated as being responsible for the biogenesis of EVs. Syntenin-1 is a cytosolic adaptor that interacts with Alix through an LYPX_n_L motif, which functions similarly to the viral late domains (22, 36, 37). LMP1 structurally does not harbor motifs that resemble viral late domains in its sequence. However, LMP1 could be sorted into these pathways through interactions with other proteins that contain L domains like Syntenin-1. Our laboratory has previously shown that LMP1 interacts with both Syntenin-1 and CD63 (26). CD63 plays an important role in LMP1 trafficking to EVs and LMP1-mediated increases in vesicle secretion (7). CD63 knockout by CRISPR/Cas9 impairs the sorting and secretion of LMP1 into EVs (7). These data suggest that LMP1 might be recruiting both CD63 and Syntenin-1 for efficient trafficking and secretion into EVs. Syntenin-1 has been suggested to be the major regulator of the CD63–Alix–Syntenin-1 complex (62). We postulate that LMP1 might be regulating the CD63–Alix–Syntenin-1 complex through Syntenin-1, which is transiently phosphorylated at Tyr4 by SRC kinase, and this activation event leads to increases in EV biogenesis and secretion (44). LMP1 can activate SRC kinase, which results in downstream PI3K/Akt activation (63, 64). Our data suggest that the activation of the SRC kinase by LMP1 may be required to phosphorylate Syntenin-1 and enhance EV biogenesis, as knocking down these proteins resulted in reduced EV production. Additionally, the loss of Syntenin-1 has been associated with reduced sizes of late endosomes, a failure of intraluminal vesicles to form, and the maturity of the endosomes. These data complement our observations that knocking down Syntenin-1 reduced LMP1 colocalization with the late endosomal marker Rab7.

The ESCRT-III complex plays a critical role in EV biogenesis and secretion, viral infection, and budding of nascent virions (42, 43, 65). Different components of the ESCRT-III complex have been demonstrated to be required for EV biogenesis and the secretion of cargo (20, 22, 47). The ESCRT-III complex is composed of CHMP6, CHMP4, CHMP3, and CHMP2, which are the core components and are thought to be required for oligomerization and scission of intraluminal vesicles (14, 35, 41, 66). Our data revealed that knocking down the CHMP4B, CHMP5, and CHMP6 subunits using shRNA impaired LMP1 packaging and secretion into EVs. These results suggest that LMP1 utilizes the canonical pathway where CHMP6 recruits CHMP4 for oligomer formation and might not be able to circumvent the depletion of CHMP6 and CHMP4A to be efficiently packaged and secreted into EVs because these core subunits play a vital role in establishing the scission complex. Once CHMP6 recruits CHMP4, this core is responsible for recruiting VPS4, CHMP2, and CHMP3, finalizing the formation of the scission complex leading to the budding of the vesicles. Although the expression of dominant negative constructs against the ESCRT-III components can arrest HIV-1 virion release from cells, LMP1 packaging and secretion into EVs were not impaired, suggesting a different mechanism of LMP1 EV incorporation (67–69). Overall, these data reveal distinct ESCRT-III subunits that drive LMP1 EV incorporation in the absence of other subunits and independent of VPS4 function. Similarly, VPS4B depletion did not alter CD63 and MHCII EV secretion, further supporting a unique ESCRT-dependent pathway that does not rely on VPS4 activity (20).

Previous studies have shown that the overall effect of inhibiting EV secretion also reduces the EV cargo being packaged. In the case of LMP1, different cargos have been found to be packaged into EVs, including EGF receptor (EGFR), fibroblast growth factor 2 (FGF-2), PI3K, and HIF1α, which play major roles in angiogenesis, tumor growth, and metastasis (9, 11, 70). Previous proteomics experiments with EBV, Kaposi’s sarcoma-associated herpesvirus (KSHV), and dually infected B cells revealed virus-specific changes in EV protein cargo, with many of the protein changes correlating with LMP1 expression in EV-producing cells. Bioinformatic analyses of these changes showed enrichments in proteins involved with immune responses, cellular movement, cell-to-cell signaling, cell death and survival, cellular growth and proliferation, actin cytoskeleton signaling, integrin signaling, as well as others (71). More recent data from Baglio and colleagues further support that EBV and LMP1 alter EV cargo (72). In that study, the proteomes of EBV-infected lymphoblastoid cell lines (LCLs) and EVs from CD40L-driven B cells were compared. EBV-specific changes in EV cargo were enriched in immune response terms, integrin-mediated signaling, and cell adhesion. LMP1-induced proteins were also enhanced in LCL EVs, including EBI2/GPR183, STAT1, and CD48/BLAST-1 protein. These data, combined with the results presented here, highlight the important roles that LMP1-modified EVs play in tumor progression since limiting the secretion of these EVs reduced cell proliferation and migration and tumor growth. Previous studies have shown that the abrogation of LMP1 trafficking to EVs leads to intracellular signaling overstimulation, resulting in reduced cell survival and colony formation (7, 8, 38). Similarly, Syntenin-1 depletion in cells might be enhancing LMP1-mediated intracellular signaling, compromising tumor growth. Alternatively, the knockdown of Syntenin-1 or Hrs could also affect the trafficking of different molecules or soluble factors, which might lead to the observed phenotypes. Altogether, our results begin to unravel the mechanisms that are required for LMP1 to be trafficked to EVs. These data also show the integral role of the ESCRT pathway and associated proteins in LMP1 trafficking and secretion into EVs similarly to how the ESCRT proteins are involved in the budding of retroviruses and other enveloped viruses. The hijacking of host extracellular pathways by LMP1 may contribute to EBV persistence or evasion of host immunity and in EBV-associated cancer may impact cancer progression.

## MATERIALS AND METHODS

### Cell culture.

HEK293 cells (ATTC CLR-1573) were grown in Dulbecco modified Eagle medium (DMEM) (catalog number 12-604Q; Lonza). The medium was supplemented with a 10% final concentration of fetal bovine serum (FBS) (catalog number 1400-500; Seradigm), 2 mM l-glutamine (catalog number 25-005-Cl; Corning), 100 IU penicillin-streptomycin (catalog number 30-002-CI; Corning), and 100 μg/ml antibiotic–0.25 μg/ml antimycotic (catalog number 30-004-CI; Corning). HK1 cells were grown in RPMI 1640 cell culture medium (catalog number 12-702Q; Lonza) with the corresponding supplements added. The cells were maintained at 37°C with 5% CO_2_. HEK293 cells were transfected with plasmids using Lipofectamine 3000 transfection reagent (catalog number L3000015; Invitrogen) according to the manufacturer’s instructions.

### DNA constructs.

GFP-LMP1 and SNAP-LMP1 constructs were previously described (19). DsRed-Rab7 WT (Addgene plasmid 12661) was a gift from Richard Pagano. Dominant negative constructs against the ESCRT III subunits CHMP2A, CHMP3, CHMP4A, and CHMP4B and the VPS4A constructs VPS4A WT and VPS4A-E228Q (gift from Nicholas J. Buchkovich, Penn State) were previously described (43).

Lentiviral shRNA constructs (Mission shRNA) for ARF6 (catalog number, TRCN0000380270), SRC (TRCN0000038150), CHMP1A (TRCN0000250119), CHMP2A (TRCN0000122479), CHMP2B (TRCN0000129922), CHMP3 (TRCN0000149440), CHMP4B (TRCN0000180330), CHMP5 (TRCN0000163206), and CHMP6 (TRCN0000151037) were purchased from Sigma-Aldrich.

The following oligonucleotides were used to construct the shRNA vectors directed against Alix, Hrs, TSG101, and Syntentin-1, where the Hrs, TSG101, Syntenin-1, and Alix target sequences were obtained from the Broad Institute GPP Web portal following searches with the corresponding gene names, and the scramble sequence was obtained by inputting the Syntenin-1 sequence into an online shRNA scramble tool through InvivoGen: GATCCCGCATAATCAAGGCACTGTAAAGTGTGCTGTCCTTTACAGTGCCTTGATTATGCTTTTTGGAAA and AGCTTTTCCAAAAAGCATAATCAAGGCACTGTAAAGGACAGCACACTTTACAGTGCCTTGATTATGCGG for Alix, GATCCCTATAGCATACTTGCATCTTTAGTGTGCTGTCCTAAAGATGCAAGTATGCTATATTTTTGGAAA and AGCTTTTCCAAAAATATAGCATACTTGCATCTTTAGGACAGCACACAAAGATGCAAGTATGCTATAGG for Syntenin-1, GATCCCGAGCTTCGCGATCCAAGATAAGTGTGCTGTCCTTATCTTGGATCGCGAAGCTCTTTTTGGAAA and AGCTTTTCCAAAAAGAGCTTCGCGATCCAAGATAAGGACAGCACACTTATCTTGGATCGCGAAGCTCGG for scramble, GATCCCGTACGTCTTCTGTCCCGTAAAGTGTGCTGTCCTTTACGGGACAGAAGACGTACTTTTTGGAAA and AGCTTTTCCAAAAAGTACGTCTTCTGTCCCGTAAAGGACAGCACACTTTACGGGACAGAAGACGTACGG for TSG101, and GATCCCCTCACGTCCGGAGTAACACTAGTGTGCTGTCCTAGTGTTACTCCGGACGTGAGTTTTTGGAAA and AGCTTTTCCAAAAACTCACGTCCGGAGTAACACTAGGACAGCACACTAGTGTTACTCCGGACGTGAGGG for Hrs.

These oligonucleotides were annealed and ligated using T4 ligase between the BglII/HindIII sites of pENTR/pTER^+^ (Addgene plasmid 17453, a gift from Eric Campeau and Paul Kaufman). The entry clones were verified by sequencing and then recombined with the destination vector pLenti X1 Zeo Dest (Addgene plasmid 17299, a gift from Eric Campeau and Paul Kaufman) using LR recombination (catalog number 11791-020; Invitrogen) according to the manufacturer’s instructions.

### Generation of lentiviral particles and cell lines.

Retroviral particles for pQCXP GFP LMP1 were previously described (7, 57). The generation of lentiviral particles for CHMP2A, CHMP3, CHMP4A, CHMP4B, VPS4A WT, GFP, and VPS4A-E228Q were also described previously (43). To make shRNA, lentiviral stocks for expression plasmids (pLenti X1 Syntenin-1 shRNA, pLenti X1 Alix shRNA, pLenti X1 scramble shRNA, pLenti X1 Hrs shRNA, or pLenti X1 TSG101 shRNA) were transfected in HEK293T cells together with packaging plasmids pMD2.G (Addgene plasmid 12259), pMDLgpRRE (Addgene plasmid 12251), and pRSVRev (Addgene plasmid 12253) to produce retroviral particles for transduction. All packaging plasmids were gifts from Didier Trono. Medium was collected at 48 and 72 h posttransfection, centrifuged for 10 min at 1,000 × *g*, filtered through a 0.45-μm filter, and frozen at −80°C until use.

Lentiviral production for the Mission shRNAs (ARF6, SRC, CHMP1A, CHMP2A, CHMP2B, CHMP3, CHMP4B, CHMP5, and CHMP6) was done using the RNAi Consortium (TRC) Broad Institute protocol (https://portals.broadinstitute.org/gpp/public/resources/protocols). HEK293T cells were transfected with the different Mission shRNAs and packaging plasmids pMD2.G (Addgene plasmid 12259, a gift from Didier Trono) and PSPAX2 (Addgene plasmid 12260, a gift from Didier Trono) using polyethylenimine to produce the viral particles. Medium was collected and stored as described above.

To make stably inducible cell lines, HEK293 or HK1 cells were initially transduced with lentivirus particles containing pLenti CMV TetR BLAST (Addgene plasmid 17492) followed by blasticidin selection (10 μg/ml) (ant-bl-1; InvivoGen). The produced stable cells were transduced again with viral particles (pLenti X1 Syntenin-1 shRNA, pLenti X1 Alix shRNA, pLenti X1 scramble shRNA, pLenti X1 Hrs shRNA, or pLenti X1 TSG101 shRNA). Blasticidin and zeocin (100 μg/ml) were used for selection of the created cell lines. Expression of the shRNAs was done using doxycycline (catalog number D3447; Sigma) to a final concentration of 1 μg/ml for 24 h before transfection with GFP-LMP1.

To make stable cell lines expressing the Mission shRNAs (ARF6, SRC, CHMP1A, CHMP2A, CHMP2B, CHMP3, CHMP4B, CHMP5, and CHMP6), HEK293 cells were transduced with lentiviral particles. The transduced cell lines were then passaged under puromycin selection at a final concentration of 2 μg/ml.

### Real-time quantitative PCR and data analysis.

Total RNA from cells or EVs was isolated by using TRIzol (catalog number 15596018; Invitrogen) according to the manufacturer’s manual. One microgram of total RNA was used for reverse transcription using qScript cDNA SuperMix (catalog number 95048; QuantaBio) in a 20-μl reaction mixture and stored at −20°C until use.

A standard 3-step cycle protocol (40 cycles of 95°C for 5 s, 60°C for 10 s, and 72°C for 20 s) was used for all qPCRs (Table 1). PerfeCTa SYBR green FastMix (catalog number 95072; QuantaBio), assay primers, and cDNA of cells or EVs were prepared in a 20-μl reaction mixture and run on a CFX96 qPCR machine (Bio-Rad). Gene expression levels were first normalized to glyceraldehyde-3-phosphate dehydrogenase (GAPDH) levels and then calculated with the ΔΔ*C_T_* method.

**TABLE 1:** qPCR primer sequences

Gene	Sequence	PrimerBank ID^*a*^
CD63	CAGTGGTCATCATCGCAGTG	91199544c1
	ATCGAAGCAGTGTGGTTGTTT	

CD9	TTCCTCTTGGTGATATTCGCCA	319738657c2
	AGTTCAACGCATAGTGGATGG	

CD81	TTCCACGAGACGCTTGACTG	62240999c2
	CCCGAGGGACACAAATTGTTC	

Alix	ATCGCTGCTAAACATTACCAGTT	371875333c2
	AGGGTCCCAACAGTATCTGGA	

Syntenin-1	TGGCTCCTGTAACTGGTAATGA	55749522c2
	CTCAGACCAACCAATGAGGCT	

Hrs	AACGACAAGAACCCACACGTC	315138978c2
	GGCCTGGATCAGGTACAGGA	

TSG101	ATGGCTACTGGACACATACCC	332000018c2
	GCGGATAGGATGCCGAAATAG	

CHMP1A	GTGTATGCCGAGAACGCCAT	379139237c1
	TTGGAGGCCACTGCGTCTA	

CHMP2A	CGCGAGCGACAGAAACTAGAG	38372936c1
	CCCGCATCAATACAAACTTGC	

CHMP2B	CATCTTTGACGGTTCTGATGACG	170650589c2
	CCTTGAGTTGCCGTTCAATCTC	

CHMP3	AGGCTGTGAGCAAGCTGTATG	7706353a3
	ATGGTGGCCTGAATCTCTGGA	

CHMP4B	TGCAGAGGAGATTTCAACAGC	260898772c2
	TGTTTCGGGTCCACTGATTTC	

CHMP4C	ACTCAGATTGATGGCACACTTTC	62526041c3
	GCTGCAAAGCCCATGTTCC	

CHMP5	GACACCAAGACCACGGTTGAT	306966145c2
	GGGTGCCATAACTGCGACTC	

CHMP6	TTGAGTTCACCCAGATCGAAATG	189409148c2
	TGGCAGCTCTATTTGTTCCTG	

a See reference 28.

### NF-κB luciferase cell reporter assay.

pHAGE lenti-NF-κB-luc-GFP has been previously described (73). The lentiviral particles were generated by transfecting HEK293T cells together with packaging plasmids as previously described (26). The lentiviral particles were used to transduce HEK293 cells expressing the different shRNAs (Alix, Hrs, Syntenin-1, TSG101, and scramble) to generate stable cell lines. The subsequent stable cells were selected using medium containing puromycin (2 μg/ml) for 2 weeks.

The generated cells expressing the NF-κB luciferase cell reporter were seeded into a 24-well plate at 10^5^ cells per well. At 24 h postseeding, the cells were transfected with SNAP-LMP1 or not transfected at all, and the medium was changed to serum-free medium. Cell lysates were harvested at 24 h posttransfection. A dual-luciferase reporter assay system (catalog number E1910; Promega) was used. Passive cell lysis was done according to the manufacturer’s protocol. This was followed by reading the assay on a luminometer according to the manufacturer’s protocol.

### Extracellular vesicle isolation.

EVs were harvested from the cells 48 h after transfection with LMP1. The collected EVs were enriched using the ExtraPEG method, as previously described (74). Briefly, the medium was centrifuged at 500 × *g* for 5 min and at 2,000 × *g* for 10 min in an Eppendorf 5804R centrifuge using an S-4-104 rotor, followed by 10,000 × *g* for 30 min in an Eppendorf 5804R centrifuge using an FA-45-630 rotor to remove cells and cellular debris. Subsequently, a 1:1 volume of 16% (2×) polyethylene glycol (average *M*_n_, 6,000) (catalog number 25322-68-3; Alfa Aesar) and 1 M sodium chloride was added to the samples, and the samples were incubated overnight at 4°C. The incubated samples were centrifuged at 3,214 × *g* for 1 h in an S-4-104 rotor. The pellet was then washed with 1× phosphate-buffered saline (PBS) and centrifuged at 100,000 × *g* for 70 min in a Beckman Max-E centrifuge using a TLA120.2 rotor. The collected EV samples were resuspended in particle-free PBS for nitrilotriacetic acid (NTA) or resuspended in 2× Laemmli sample buffer (4% SDS, 100 mM Tris [pH 6.8], 0.4 mg/ml bromophenol blue, 0.2 M dithiothreitol [DTT], 20% glycerol, 2% β-mercaptoethanol [BME]) for immunoblot analysis.

### Nanoparticle tracking analysis.

Nanoparticle tracking was performed using a Malvern NanoSight LM10 instrument, and videos were processed using NTA 3.4 software as previously described (7, 75).

### Immunoblot analysis.

Whole-cell lysates were harvested at 48 h posttransfection, centrifuged at 500 × *g* for 5 min to collect cell pellets, and lysed using radioimmunoprecipitation assay (RIPA) buffer as described previously (7, 57). The cell lysates were centrifuged at 22,220 × *g* for 10 min at 4°C to remove insoluble material. The lysates were mixed with 5× Laemmli sample buffer (10% SDS, 250 mM Tris [pH 6.8], 1 mg/ml bromophenol blue, 0.5 M DTT, 50% glycerol, 5% BME) to a final concentration of 1× and boiled at 95°C for 10 min. An equal amount of protein was loaded onto an SDS-10% PAGE gel for electrophoresis and then transferred to a nitrocellulose membrane. The blots were blocked in a Tris-buffered saline solution containing 0.1% Tween 20 (TBS-T) and 5% nonfat dry milk. The primary antibodies used included antibodies for Alix (clone Q-19; Santa Cruz), HSC70 (clone B-6; Santa Cruz), TSG101 (clone C-2; Santa Cruz), CD81 (catalog number sc-9158; Santa Cruz), CD9, Syntenin-1 (catalog number sc-100336; Santa Cruz), Hrs (catalog number A300-989A; Bethyl), ARF6 (catalog number 5740s; Cell Signaling), c-SRC (catalog number sc-8056; Santa Cruz), GFP (catalog number 600-101-215; Rockland), Flotillin-2 (clone H-90; Santa Cruz), CD63 (clone TS63; Abcam), calnexin (clone H-70; Santa Cruz), LMP1 (clone CS1-4; Dako), and SNAP (catalog number P9310S; NEB). The blots were subsequently incubated with the following horseradish peroxidase (HRP)-conjugated secondary antibodies: rabbit anti-mouse IgG (catalog number 26728; Genetex), rabbit anti-goat IgG (catalog number 26741; Genetex), goat anti-rabbit IgG (Fab fragment) (catalog number 27171; Genetex), and anti-mouse kappa light chain (clone H139-52.1; Abcam). Following four TBS-T wash steps (5 min each), the blots were incubated with Pico ECL (catalog number 34080; Thermo). The blots were then imaged using an ImageQuant LAS4000 imager (General Electric) and processed with ImageQuant TL v8.1.0.0 software, Adobe Photoshop CS6, and CorelDraw Graphic Suite X5.

### Confocal microscopy.

HEK293 cells expressing scramble, Hrs, and Syntenin-1 shRNAs were seeded into 35-mm glass-bottom plates (catalog number 627860; Greiner Bio-One) for live-cell confocal microscopy and induced with doxycycline (1-μg/ml final concentration) at 24 h postseeding. At 48 h postseeding, cells were either transfected with GFP or GFP-LMP1 or cotransfected with GFP-LMP1 and DsRed-Rab7 WT. Nuclei were labeled with Hoechst 33342 dye (catalog number 62249; Thermo Scientific) to a final concentration of 5 μg/ml according to the manufacturer’s instructions. A lysosomal stain (Lysotracker red DND-99, catalog number L7528; Invitrogen) was added to cells for 15 min to a final concentration of 50 nM before the medium was changed. Images were taken using a Zeiss LSM 880 microscope with 488-nm and 594-nm lasers and processed using Zen 2.1 Black software.

### EGF uptake and degradation.

HEK293 cells expressing the ESCRT-III dominant negative constructs (GFP, CHMP2A, CHMP3, CHMP4C, and CHMP6) were seeded in the presence of doxycycline in 35-mm glass-bottom plates. At 24 h postseeding, the cells were kept on ice for 10 min, followed by washing with a live-cell imaging solution (LCIS) containing 20 mM glucose and 1% bovine serum albumin (BSA). Texas Red EGF complex (catalog number E3480; Thermo Fisher) was added to the prepared LCIS to a final concentration of 2 μg/ml, and the mixture was incubated at 37°C for about 15 min. The cells were washed three times with LCIS, and nuclear staining was also done. Images were taken on a Keyence BZ-X710 fluorescence microscope.

### Cell proliferation and migration assays.

Cell proliferation and migration were measured using the xCelligence RTCA DP instrument (ACEA Biosciences, San Diego, CA, USA). For cell proliferation, HK1 cells expressing LMP1 and the shRNAs (scramble, Hrs, and Syntenin-1) were initially seeded into 10-mm plates and induced with doxycycline at 24 h postseeding. At 48 h postseeding, the cells were dissociated using trypsin, and the cells (10,000) were seeded into 16-well plates (E-16 plate, catalog number 5469830001; ACEA Biosciences) to monitor proliferation using electrical impedance. A minimum of four wells for each sample were measured. For cell migration assays, HK1 cells were also prepared described above and seeded into a CIM-16 plate. The lower chamber was filled with medium containing 1% FBS to act as a chemoattractant. The cells (40,000) were seeded into the upper chamber in serum-free medium, and readings were taken every 10 min for 24 h.

### Animal experiments.

Six-week-old female athymic nude mice (Foxn1^nu^) were purchased from Envigo. The mice were housed for about a week before beginning the experiments at the Florida Agricultural and Mechanical University animal care facility. For the subcutaneous xenograft model, HK1 cells expressing either doxycycline-induced GFP-LMP1 or syntenin-1 shRNA and GFP-LMP1 (2.5 × 10^6^ cells/animal) were subcutaneously injected into the right flank of athymic nu/nu mice. Two weeks after cell implantation, doxycycline (50 μg/g of body weight) oral treatment was given to all animals weekly. Tumor growth was measured at least every 5 days.

Animals used in experiments were housed according to regulations set by the American Association for Accreditation of Laboratory Animal Care at 37°C with 60% humidity, and procedures were modeled after methods approved by the Institutional Animal Care and Use Committee.

The experiments performed were reviewed and approved prior to being executed by the Institutional Animal Use and Care Committee of Florida Agricultural and Mechanical University (protocol number 017-03, Office of Laboratory Animal Welfare assurance number A-3581-01).

### Statistical analysis.

The significance of results was determined by Student’s two-sample *t* test and ordinary one-way analysis of variance (ANOVA). Figures were assembled by using the Microsoft Excel, Adobe Photoshop CC2019, GraphPad 8.3, and CorelDraw 2019 software programs.
